# A Qualitative Exploration of Patient Activation Among Hispanic Breast Cancer Patients

**DOI:** 10.21203/rs.3.rs-9869309/v1

**Published:** 2026-07-29

**Authors:** Ingrid Zeledon, Timothy J. Grigsby, Jennifer B. Unger, Gabriel Luna, Daniel W. Soto, Tess Boley Cruz

**Affiliations:** University of Southern California; University of Nevada, Las Vegas; University of Southern California; University of Southern California; University of Southern California; University of Southern California

**Keywords:** Patient Activation, Hispanic/Latino, Breast Cancer, Qualitative Interviews, Familism, Respeto

## Abstract

**Objective:**

Patient Activation is a patient’s knowledge, skill, and confidence for self-management of health- is positively correlated with medical regimen adherence and improved health outcomes. Understanding patient activation in Hispanic women diagnosed with breast cancer—conceptualized in the context of Latino cultural values of *familism and respeto* is essential to develop sustainable and culturally appropriate structural and individual interventions.

**Methods:**

In-depth semi-structured interviews were conducted with ten Hispanic women who were recently diagnosed with breast cancer or undergoing treatment. Transcripts were analyzed using thematic analysis, whereby open coding was applied to identify recurring patterns, which were subsequently organized into meaningful themes reflecting participants’ lived experiences.

**Results:**

Family support emerged as a crucial factor for maintaining high treatment involvement, especially when family members served as surrogate information seekers and contributed to medical decisions. While family support encouraged adherence to treatment, it also reduced women’s expression of adverse effects related to cancer treatment. Elements of patient-provider interactions that facilitated patient engagement in treatment included physicians speaking the same language or having the same cultural background as the patient, encouraging questions, and having a calm demeanor.

**Practice Implications:**

Interventions to facilitate patient activation, especially in the absence of social support, could be useful for Hispanic women throughout the course of cancer treatment.

## Introduction

Breast cancer is the leading cause of cancer incidence and mortality among Hispanic women (1, 17). Healthcare underutilization contributes to this trend; Hispanics are more likely than non-Hispanic Whites to experience late detection, treatment delays, and mortality (2, 3, 18, 19). Barriers to treatment exist at multiple levels including intrapersonal barriers (e.g., low health literacy, low understanding of the healthcare system, and cultural values or beliefs that dissuade them from seeking Western treatment); interpersonal barriers (e.g., patient-provider interaction); and institutional barriers (e.g., lack of insurance and lack of access to culturally competent care) (4, 31, 40).

Cancer care is a complex process that involves patient engagement in primary prevention, detection, diagnosis, treatment, and post-treatment survivorship or end of life care (5, 6). Navigating the healthcare system requires sufficient health literacy and patient activation to advocate for one’s needs, understand treatment regimens, and make informed choices. Patient activation involves the development of the skills, knowledge, and beliefs necessary to take an active role in personal healthcare (7). It involves fourstages in which patients believe an active role is important, have confidence and knowledge to take action, take action, and (17) persist under stress.

There are benefits to improving patient activation, it is associated with appropriate use of the healthcare system, self-management of chronic diseases, and healthy behaviors (34, 35). Importantly, it is modifiable (23, 34). Brief in-person and online interventions have improved patient activation in breast screening behaviors and mental health among Hispanics (8, 24).

Interpersonal and cultural factors can be facilitators or barriers to patient activation (37). Previous studies have reported *respeto* (not questioning authority figures) increases Hispanics reluctance to request clarification of physicians’ instructions or second opinions. *Familism* emphasizes family interconnectedness, fulfilling familial obligations, providing support, and subjugation of self for family (9, 20, 32, 33, 36). This cultural value also shapes the perceived role of women within the family unit as a source of strength and unity for the family (33). For women who are accustomed to fulfilling other family members’ needs, familism might make it difficult to seek care for their own needs. Conversely, familism is protective against loneliness, depression, and physical symptoms and positively associated with selfesteem and subjective health (10). Additionally, familism influences health behaviors such as including family members in medical decision making or seeking health information on behalf of family members (20).

Patient-provider communication is another interpersonal factor impacting patient activation. Physician-patient concordance (sharing the same language, ethnicity, communication style or gender) and patient demographics can interact to create a patient-provider relationship that is conducive to patient activation (11, 21, 22). Patients perceive race-concordant physicians to be more collaborative in navigating patients through medical decision making, which is critical as Hispanics often report apprehension about asking questions for fear of threatening the patient- provider relationship (37).

While patient activation has been applied to cancer screening, prevention, and survivorship (12, 35), less research has focused on patient activation during the cancer treatment experience (25, 26), when patient activation might be most critical for improving health outcomes. Despite the well-documented influence of cultural values such as familism and respeto on health behaviors, their specific role in shaping treatment engagement and patient activation among Hispanic women diagnosed with breast cancer remains understudied. To address this gap, thematic analysis of in-depth semi-structured interviews was used to explore the lived experiences of ten Hispanic women recently diagnosed with or undergoing treatment for breast cancer, with the aim of identifying culturally congruent beliefs and practices associated with high patient activation. Findings from this exploratory study have implications for both patient and clinician education, contributing to more culturally responsive and equitable care across the cancer care continuum.

## Methods

### Participants and recruitment

Participants were approached in the waiting room of the breast cancer clinic at Los Angeles County + University of Southern California Medical Center (LAC + USC), a public County-run teaching hospital that provides services to underserved communities. Patients seen at LAC + USC are covered by emergency Medicaid or other low-cost programs offered by the hospital. Women were screened and included in the study if they were Hispanic and had been diagnosed within the last year with breast cancer but had not started treatment or were previously diagnosed and still in cancer treatment. Demographic information was not collected to ensure patient privacy. All materials (e.g. interview tools, consent forms) and process were approved by the USC Institutional of Review Board (IRB). Women who met these criteria were invited to participate in a semi-structured interview about their breast cancer treatment experience. Participants were briefed about the study and signed informed consents. Interviews were conducted in a private room in Spanish, and participants received a $20 incentive gift card. Ten women met the inclusion criteria and completed the interview.

## Data collection and analysis

Individual semi-structured interviews included open-ended questions exploring patient-provider interactions and the treatment process (e.g., *‘Describe some of the main barriers that make communication about your disease with medical professionals difficult’* and *‘Describe how confident you are in understanding what medical personnel tell you regarding your disease’*). Interviews were audio-recorded, transcribed verbatim, and translated into English prior to qualitative analysis. NVivo software was used to organize and manage transcript data.

Transcripts were analyzed using thematic analysis. Open coding was first applied to each transcript to identify initial codes reflecting patterns of patient activation across stages of the treatment experience. To build the codebook, three interviews were coded collaboratively by two coders, with codes emerging from ongoing discussion and iterative refinement. Discrepancies were resolved through consensus, and the codebook was finalized following this process. The remaining interviews were then systematically coded by the lead author using the established codebook.

## Results

All participants reported engaging support from others, and most utilized online resources or surrogate information seeking. Among women with children, familism was a prominent theme, particularly the belief in needing to serve as a source of strength for their families, alongside concerns about how their diagnosis would affect their role as matriarch. Respeto was expressed primarily through participants’ perception of physicians as the ultimate authority on treatment decisions and was reflected in high levels of treatment adherence.

Additional themes emerged around strategies participants used to remain engaged in their treatment as well as barriers to health care access. Engagement strategies included: asking questions of providers; seeking information online or through family members; using alternative medicines recommended by friends and family; and (17) strictly adhering to prescribed medical treatment. Women reported asking questions either directly to providers or through the use of translators. Notably, participants did not describe taking an active role in their care through seeking a second opinion or expressing disagreement with physician-recommended treatment plans. Barriers to health care access included limited availability of translation services, concerns about insurance coverage, and lack of transportation (See Table 1 *Examples of Active Treatment Engagement Through Social Support and Family Networks)*.

## Disclosing diagnosis to family as a form of activation & the role of Familism

Participants’ active management of their own health frequently involved engaging others to assist in their care. At the time of diagnosis, many participants described a period of indecision regarding whether to disclose their diagnosis to family members. The initial inclination for many was to conceal their diagnosis in order to protect loved ones from worry or emotional pain. This was especially true among the nine participants who had children, who expressed a strong desire to shield them from the distress of the diagnosis.

The decision to withhold disclosure was rooted in familism values, particularly the cultural expectation that women serve as the primary source of strength and unity within the family. However, all participants ultimately disclosed their diagnosis to family members who could provide support throughout their treatment (see Table 2 for examples of seeking social support and relying on extended family networks) Familism also shaped participants’ eventual decisions to disclose, as accepting help from family members was motivated by the long-term desire to heal and remain present for their families.

It is worth noting that while the Patient Activation Measure (PAM) defines active role-taking largely through indicators of self-reliance, the nature of a cancer diagnosis reframed what active engagement meant for these participants. Rather than independence, active management often meant deliberately seeking social support and engaging family networks. As such, the decision to disclose their diagnosis can itself be understood as a form of active participation in their treatment, as it consistently served as a pathway to mobilizing social support.

## Information Seeking & Alternative Medicine

Participants expressed a strong desire to understand their diagnosis and treatment options, engaging in active information seeking through multiple channels. This included asking questions of their providers, searching for information online, and relying on family members who conducted searches on their behalf. Frequently, online health information searches led participants to complementary and alternative medicine (CAM) — products and interventions that fall outside the scope of standard medical care.

Participants described the use of alternative medicine as a complementary strategy alongside conventional cancer treatment rather than a replacement for it. Approaches to integrating CAM varied among participants. Some consulted their physicians prior to initiating an alternative treatment, while others self-medicated without disclosing their use of CAM to their providers. The decision not to disclose was largely driven by fear that their physicians would be dismissive of or unsympathetic to alternative medicine practices. From the participants’ perspective, engaging in information seeking and utilizing alternative medicine represented a meaningful form of active participation in their own treatment process (see Table 2 Participant Strategies for Information Seeking and Use of Complementary and Alternative).

### Provider Communication & Respeto

Most participants preferred physicians who spoke their native language, as even when translators were available, fully understanding medical information remained challenging. Participants also preferred direct communication regarding their diagnosis, delivered in plain language with clear explanations of the steps involved in their treatment.

Participants held strong expectations for physician-initiated communication throughout the treatment process. They expected their physicians to provide a comprehensive overview of their treatment course, offer regular progress updates on their overall health, and proactively share recommendations regarding diet and exercise — without requiring participants to prompt these conversations. Consistent with the cultural value of respeto, participants deferred to their physicians as the experts on their condition, trusting them to guide and direct their care. Participants looked to their physicians to be proactive in providing clear, timely information on their progress and next steps (see Table 3 Respeto and Provider Communication Among Hispanic Women with Breast Cancer).

## Medical Decision Making & Familism as a Facilitator of Medical Adherence

At key decision points in treatment, most participants preferred to defer medical decision-making to their physician or to consult with a family member before reaching a decision. Participants generally expressed a strong desire to begin treatment immediately and reported high levels of trust in their physicians’ recommendations. Family members played a dual role in participants’ treatment experiences, providing both emotional support and practical assistance in navigating the healthcare system. This included accompanying participants to medical appointments, engaging directly with physicians, and asking questions regarding participants’ progress and care. In many cases, participants also relied on family members to assist in making medical decisions.

Family members consistently reinforced treatment adherence and, in some instances, actively discouraged participants from disagreeing with physician recommendations. While this dynamic facilitated compliance with prescribed treatment plans, it also presented limitations. In rare cases, deference to family pressure prevented participants from providing candid feedback to their physicians regarding treatment symptoms or adverse experiences, potentially compromising the quality of their care (see Table 4 Familism as a Driver of Medical Decision-Making and Treatment Adherence Among Hispanic Women with Breast Cancer).

## Access to Healthcare as a barrier to patient activation

Because participants were recruited from the LAC + USC Health Center, all had some form of insurance coverage, most commonly Medicaid. LAC + USC also offered a range of wrap-around support programs, including nutrition and exercise classes and a complimentary wig program for women experiencing hair loss as a result of treatment. All participants were aware of these additional programs, and many had utilized them.

Notably, while participants had the option of seeking a second opinion, none did so. It remains unclear whether this decision reflected a perceived or actual barrier to access. Given that many participants were enrolled in Medicaid or other limited coverage plans, it is possible that they believed second opinions were not covered or available to them, or that they felt they lacked the agency to select or change their physician. Consequently, neither seeking a second opinion nor changing physicians emerged as a strategy participants identified as a form of active participation in their care.

The following excerpt represents the only instance in which a participant described contemplating seeking a second opinion:

**P4: “**If I am unhappy, I say you know what, I am not happy, can you refer me [to another physician] and they do. Sometimes they call and they make an appointment and they say, ‘ok they’re [the referred physician is] going to call you’ and they do call you and I say ok but I don’t want to leave you, I just want to keep you and I still want you to look at my situation, but can you refer me [for a second opinion]. But then sometimes if I ask to go to another doctor, they will say ‘if you’re going to leave, you have to let us know and then they’re not going to take you back. If you’re going to leave this hospital, then they’re not going to take you back.’ And sometimes that makes me a little bit unhappy because sometimes I want a second opinion.”

### Emotional Overwhelm and Treatment Engagement

Most participants reported experiencing periods of emotional overwhelm during their treatment that temporarily impaired their ability to fully process medical information. This emotional distress was largely transient, occurring most commonly during the initial period following diagnosis or at critical junctures when treatment status changed. Once participants had processed and come to terms with their diagnosis or disease progression, they reported being able to re-engage actively in their treatment. The presence of a support person during medical appointments proved particularly valuable during these periods of heightened emotional distress. Having a trusted companion accompany them helped participants process information and formulate questions about their next steps in treatment — especially at moments when emotional overwhelm made independent decision-making and information processing difficult (See Table 5 Social Support as a Coping Strategy for Treatment Transitions and Emotional Stress Among Hispanic Women with Breast Cancer).

## Discussion

Patient activation is influenced by cultural beliefs, patient-provider interactions, and accessibility to credible resources and information (15, 21, 22, 39). In the present study, emotional stress was reported to interfere with participants’ ability to process information and engage in medical decision-making. This finding is consistent with previous research demonstrating that patient activation is influenced by the perceived course of illness and changes in self-rated health, and that it can fluctuate over the course of treatment (26, 30).

Transitions between phases of the cancer care continuum have been categorized as either personal transitions — encompassing physical, emotional, or social changes — or care transitions, which include changes in cancer status, treatment, and approach to care (38). Participants in the present sample primarily described emotional transitions experienced throughout the course of their treatment. To navigate shifts in mood and motivation, participants focused on self-care activities and acceptance of their diagnosis.

Current interventions tend to target patients either post-treatment during survivorship or at discrete transitional periods, and are typically formatted as a one-time module. However, given the fluctuations in emotional stress and cognitive overload reported by participants throughout the course of treatment, future interventions should incorporate components designed to support patients in coping with emotional transitions on an as-needed basis or continuously throughout the entire treatment course.

Most Hispanic women in this study described familism as a protective cultural factor in their cancer experience. The facet of familism that positions women as the primary source of strength within the household generated significant indecision among participants regarding whether to disclose or conceal their diagnosis from family members. While disclosure offered access to social support and aligned with participants’ long-term desire to heal and remain present for their families, many were reluctant to burden or worry their loved ones. Ultimately, all participants chose to disclose, and familism served as a mechanism for coping with emotional stress by engaging spouses or parents in the medical decision-making process. Tangible support was frequently provided by extended family members throughout treatment, including childcare and meal preparation. Surrogate information seeking among friends and family members represented an additional form of tangible support. Current interventions that provide patient activation guidance to both patients and family members or caregivers simultaneously remain limited, yet should be explored as a core component of cancer care interventions with Hispanic patients given these qualitative findings (25).

Similarly, the cultural value of respeto contributed to higher treatment adherence and stronger rapport in patient-provider communication. While patient-centered communication styles are known to facilitate patient activation, respeto as a Latino cultural value may discourage individuals from asking questions out of fear of being perceived as disrespectful (13, 22, 37). The findings of the present study corroborate this, as participants who viewed their physicians as authoritative figures frequently reported withholding information about treatment side effects or feeling they lacked a legitimate voice to disagree with medical recommendations. Previous research has found that Hispanic patients may feel less empowered to request health information and advocate for their needs, potentially contributing to poor patient-provider communication, low satisfaction with quality of care, and reduced adherence to medical treatment regimens (14, 21). Socioeconomic status further exacerbates these challenges, as several participants expressed concern that non-adherence would result in the loss of available resources, leaving them feeling that compliance was their only option — even when experiencing adverse side effects from treatment. The family system often reinforced this compliance.

Reframing the patient-provider relationship for both the diagnosed individual and the family members who assist in medical decision-making may help capitalize on the protective aspects of respeto. For example, speaking up could be reframed not as dissent, but as providing physicians with information necessary to tailor treatment effectively. A promising strategy for improving patient activation involves counseling patients and supporting family members in understanding their rights and responsibilities in the care process. Specifically, informing patients of their right to ask questions, receive answers, request clarifications, and report new symptoms or side effects can empower them to engage more actively with their providers. Research on how to help Hispanic patients redefine their relationship with providers toward a more collaborative model remains limited. However, recent findings suggest that establishing a collaborative patient-provider relationship may be a prerequisite for improving patient activation (29). Future research should continue to explore culturally congruent approaches to fostering collaborative patient-provider relationships.

Online health information seeking was commonly reported among participants. Given the rapid expansion of eHealth and the vast volume of information available on the Internet, developing strategies to support patients in effectively navigating online health content could assist them in becoming critical consumers of information and increase the likelihood of accessing credible sources (16). For example, as part of initial treatment planning, medical staff could teach patients to identify accredited health websites or search medical databases. This approach could also be applied to surrogate information seeking, such as when friends or family members recommend alternative medicines or other health-related strategies.

The widespread reported use of alternative medicine among participants underscores the cultural significance of complementary treatment within Hispanic communities (27, 28). Greater physician awareness of alternative medicine practices can encourage more complete disclosure from patients. Individuals should be counseled to share any intentions to use alternative treatments with their physicians prior to initiation. Combined with interventions that support patients in critically evaluating online health information, patients may be more inclined to weigh empirical evidence in their treatment decision-making process. Additionally, normalizing physician consultation regarding alternative medicine use may further improve disclosure. Physicians, in turn, should actively listen to their patients, communicate that questions are welcomed, and provide culturally sensitive, evidence-based feedback to encourage ongoing disclosure and sustain a strong patient-provider relationship

## Limitations

Several limitations of the present study should be acknowledged. Participants were recruited from an institution that provided access to wrap-around care services, a level of support not commonly available across healthcare settings. As such, this sample is likely not representative of underserved communities seeking cancer care more broadly. Additionally, participants were largely low-income individuals with Medicaid coverage, and therefore may not represent the full spectrum of socioeconomic backgrounds among Hispanic women with breast cancer.

Future studies should examine whether patient activation differs by socioeconomic status, immigration status, acculturation, and education level. Information on cancer staging at the time of diagnosis was not collected in the present study; however, future research may find that disease progression interacts meaningfully with patient activation. It should also be noted that this study was not designed to capture the experiences of women in post-treatment survivorship requiring surveillance, which represents an important phase of the cancer care continuum that warrants further investigation.

Despite this limitation, the strong familism values identified among participants suggest that incorporating family support into patient activation frameworks — or developing a caregiver-specific patient activation measure — may represent a culturally congruent adaptation of existing measures for use with Hispanic populations. Future research should consider employing a mixed-methods design to capture patient activation levels quantitatively, enabling direct comparison with qualitative assessments and strengthening the validity of findings.

## Conclusions and Future Directions

Understanding patient activation within the framework of Hispanic culture represents an important step toward reducing health disparities in breast cancer outcomes. Current patient activation measures, including the PAM, primarily assess individual self-reliance and the extent to which individuals independently manage their own health. However, within the context of Hispanic culture and given the nature of a cancer diagnosis, proactive health engagement frequently involves enlisting others in one’s care rather than acting alone.

Family support is integral to the health experiences of Hispanic women, and interventions aimed at improving breast cancer outcomes in this population could benefit significantly from a family- or caregiver-inclusive approach. The PAM stages and 13-item scale focus on individual behavior in proactively seeking and processing health information; however, cultural adaptation of the PAM may be warranted to incorporate the process of engaging family or social support networks as a recognized and valued form of patient activation. Given the central role of family members in Hispanic communities, their intentional integration into the patient’s care plan has the potential to reinforce and sustain patient activation throughout the cancer care continuum.

## Figures and Tables

**Table 1:** Examples of Active Treatment Engagement Through Social Support and Family Networks

Interviewer: Describe a conversation with doctors that you felt went well.
**P2**: “I was really surprised, I became depressed because I have four adolescent daughters, they are in school, and I didn’t want them to find out because I didn’t want them to live worried, suffering because their mom was sick. But how am I going to do it? … Because I want to protect my daughters from finding out that I am sick, I don’t want to see them worried. They are studying, let them make their lives, let them be happy, I’m not dying. Well, well if it got to the point that I need chemotherapy, I’m not going to be able to hide it. Your hair falls out and who knows if it would’ve worked for me. So, I waited. When I saw that it wasn’t necessary, when I saw that the medications didn’t affect me much, that I led a normal life, then I was able to hide it. So, to be able to keep that hidden, I couldn’t tell anybody, absolutely no one from my family because, although you can trust somebody, it always gets out… **And the irony of it was that at time they diagnosed me, well I didn’t have a job, so I ultimately depended on my brothers**.”
**P3**: “My son is 18 and my daughter is 20. Like I tell you, it’s not the same when they tell you that you’re in danger because I would think- ‘what’s going to happen to my children’ when they told me the news about cancer… And I was planning on going to Mexico and I told my husband, in those days, before the second appointment where they told me I had cancer…‘you know what? I want to go to Mexico. Tell my children that I’m going to take care of my dad that he’s sick, that I’m going to be with him in Mexico.’ That I didn’t want, because since they were telling me about the symptoms, about the hair and paleness and I don’t know what else, well I started imagining it and I was [thinking] well you’re starting to kill me already, so I told my husband: ‘I’m going to leave.’ My husband was like ‘no we’re going to face this all together, don’t worry, everything will be okay.’ I know that for them with me it’s difficult, but it’s more difficult as a mother, seeing all them like that, and it’s hard it’s terrible… So, I said, my dad is already elderly, I’m going to put him through suffering, he’s going to die of sadness, it’s going to be worse instead of one [dead] it’s going to be two. No, so, that’s when I sat down [and decided to stay in the U.S.]. Then when my husband got home, we talked, he started to cry, because it’s not an easy thing…”
P8: [Family is] a big part of my process, they have always been there with me. Interviewer: You told them from the start? P8: Yes. Well my son like a month later. Interviewer: Why? P8: Because I knew he would feel bad because he is more sensitive, but my daughter the eldest, and my siblings, and my husband, they all knew in that moment.

**Table 2:** Participant Strategies for Information Seeking and Use of Complementary and Alternative Medicine

Interviewer: Describe what worked for you when you talked about your health with your doctor.
**P3**: *Asking questions. Because I think that you shouldn’t stay quiet, especially when it’s about your health. That, although sometimes the doctor, I know they have a lot of patients and all that, but also the patients are left with doubts because one doesn’t know about the body, organism, which are the medications, which are the treatments, and those things until one asks for an explanation- What can I do? How will it [treatment] initiate?*
P9: *Yes, for example I would tell him like oh look these people say that, for example what could I tell you, that she’s thrown up too much, that with the medications this, he would say okay, they are side effects that could happen, but it depends on every body, because not all bodies respond. For example, for me, my body would respond with one medication, maybe for them no, they would tell me no ask for this and ask for that. I would tell my doctor oh look they recommended this to me, he would say do you want to try? Yes. But it wouldn’t work for me. He would ask what happened and I’d say it didn’t work for me. He’d say okay it’s because not everybody is the say, I will change it for you, and like that. I would be asking for things and then for example, in chemotherapy they would ask for, they would give me a lot of medicine that I felt that they would leave me completely exhausted. But I said I have to be strong, and I have to get out of here, so I would say don’t give me this, don’t give me that, that no, I’m going to speak with the doctor. So I would say to the doctor, doctor look, this medication, I feel like it makes me too sleepy, it leaves me bad. Okay, I would like for you to stop this medication. Okay, we’ll try, we’ll stop it, if you have chemotherapy and you feel better, for the next one we won’t give it to you and we’ll go on and see like that. So he would stop some medications, don’t give me this, don’t give me that, and I would speak with the doctor and like that, they went and stopped medications*.
**Interviewer**: Are there any other people who help you understand your treatment options? Aside from the doctors.
**P1**: *“Like they tell you, oh drink bicarbonate with lemon, but don’t tell the doctors because they don’t believe. So you don’t tell that to the doctors. And you do things on the outside that I did do. So I never told the doctor and I’ve never told them.”*
**P4**: *“umm, just the doctors, no one else*…*it’s difficult, like for example, you know what I do, I research in the Internet..”*
**P3**: “…*whatever I heard, I would take. They would tell me, put on packets of chamomile for the inflammation to go down, so I would put that. Put on aloe or take a smoothie of that. If you ask me what cured me miss, I don’t know because I would take everything. Of everything. I took a juice of guanabana, natural guanabana. Green juices, even if I didn’t like them.”*

**Table 3:** Respeto and Provider Communication Among Hispanic Women with Breast Cancer

Interviewer: Describe a moment in which you felt that the conversation went will with your doctor.
**P4**: “I would like the doctors to at least speak your language and be able to give an explanation when someone has a question. Not that they speak so much that they know all the name of the nerves and what not, but for them to tell me what they’re going to do to me. Not that there could be this, there could be that, no, tell me what you’re going to do to me. Do I have a cure, or do I not have a cure? You know what I mean?”
**P3**: Ah, obviously when I spoke with the oncologist directly in my language, it made me feel more comfortable, more confident because that way I could express my concerns and he would respond. For example, the last [physician] I saw has been the best out of all of them, because there were questions, I had made prior [to that visit], that were answered, but I always left feeling like I was missing something. So, the last [physician] explained everything in depth, and I left satisfied.
**P10**: She is very open; she is easy to talk to and she speaks Spanish very well. That’s what I liked about her, her character is very [likable], everything that I asked, she knew how to answer very well, and I liked how she treated me.
**P6**: …the attention that they give me, I feel good about. Because I have heard many people who say that sometimes they don’t understand, that sometimes the doctors aren’t friendly with them and thank God that with me, yes. Always when I’ve come here, I’ve felt very good.
Interviewer: Describe a moment in which you felt that the conversation with your doctor did not go well.
**P2**: “…up to a certain extent, I’ve noticed that they are bit reserved. Because, unless I mention the things that can improve my well-being, they don’t say it. So, like, up until now, with a doctor, he’ll tell me, or an oncologist, you know that ’this diet will help you, you shouldn’t eat this, no’. But it is not until I mention it [first]. When I think it should be the other way around, right? The doctors are the experts, we as patients, sometimes don’t know almost anything. So like, it’s up to them, up to the doctors, to tell us what’s best for us.”
**P4**: “…sometimes I have to ask them if they saw my lab, my blood, because sometimes I make my own notes, and I come to ask the doctor and sometimes the doctor doesn’t know what I want to know and sometimes I have to tell him “ok check me here if I am good, how did the blood come out, um, um, and medications’ or sometimes I ask, there are some doctors that say that your hair is going to fall out, sometimes some don’t tell you, and sometimes I have to ask ‘listen doctor is this good for my body? Is my hair going to fall out, am I not going to feel well?’ and sometimes I keep asking and sometimes some doctors don’t tell you…”

**Table 4:** Familism as a Driver of Medical Decision-Making and Treatment Adherence Among Hispanic Women with Breast Cancer

Interviewer: Describe a couple of things or people (could be family members, other individuals, print materials, or other things) that help you understand conversations you had with medical professionals about your illness?
**P6**: “…I told her I understood everything [the doctor] told me, they asked me if I was okay, if I was out of it, and I said ‘No, I did understand, I just want to know when the whole process will begin, what is the first thing that is going to happen, what’s going to come next?’ Then when the doctor told me what was going to happen first, that it was going to be an operation. And through that surgery they would check to see if the cancer had spread. And so, I told her it’s all good, do it. When do I have to start? The quicker they give you the appointment, the better. Because if you have something wrong with you, what matters is removing it, you know. If they’re telling me that there’s something bad inside of me, well take it out, take it out!”
**P7**: “My family, the doctors themselves, they are who I’ve had communication with. And I have tried to be the most open in my case, to be able to get better information.” **Interviewer**: How does your family help? **P7**: “They give me a lot of support, with a lot of love, and they treat me better. Yeah, with the cancer case. That is important, that family supports you, that they don’t bring you down, that they don’t look at you badly, all those things.” **Interviewer**: Have family members come with you during your visits? **P7**: “My husband comes and he also asks questions to the doctor and he asks questions that maybe I don’t want to ask, and he asks.”
**P9**: “Oh, well my husband, he’s always helped me, because sometimes when he’s accompanied me to the appointments, sometimes they’re in English and I don’t speak a lot of English. He translates for me or he explains to me because sometimes I’m left there like I don’t manage to understand, and he explains or I tell him, ‘oh ask this to the doctor’ and he tells me and that’s when I’m left at peace and like that I can delegate [my questions] to my husbands because sometimes I’m unsure of my questions and he tells me ‘well ask the doctor, ask this’ and he helps me with things I don’t understand or go over my head, he tells me hey why don’t you ask this, [and I say] hey you’re right I hadn’t thought of that. And so, my husband helps me, my sister, or someone close to me, helps me.”
**P4**: “On Monday when I came, well, you don’t feel when they are changing the chemo, because it debilitates you, it makes you fall asleep…and then when I went home, the next day, because chemo doesn’t affect you immediately, it does it little by little… [when we arrived home] I told my mom: ‘I can only see a dark shadow and everything is white around.’ She said, ‘oh my God, no that’s not right, we have to call the doctor’…when I got here [to the hospital], my mom told the guys, ‘my daughter can’t see and she feels dizzy. Something is happening to her because she’s seeing spotty.’
**P5**: “…and sometimes I’m not too happy about it and sometimes I tell the doctor that I just want a month on and a month off [chemotherapy] because I feel like I’m not happy, I feel like I’m getting worse, instead of getting better. That’s one thing I noticed, but then when I tell that to my parents or my husband or my family, they say that I have to follow what the doctor says. I have to take what the doctor recommends, but then in my heart…I feel like it’s not good.”

**Table 5:** Social Support as a Coping Strategy for Treatment Transitions and Emotional Stress Among Hispanic Women with Breast Cancer

Interviewer: Do you have people that come with you and help you communicate with the doctors, they can be nurses, if you don’t understand something?
**P4**: No, I have always come by myself and sometimes I asked all my questions to the doctors and um I’ve never needed someone else. Only when I am really not well and sometimes I can’t um, like handle the situation that the doctor tells me because sometimes I cry when they tell me one thing, but I have family and sometimes they come and accompany me. …Yes, like the storm system, [it will] pass, you will go through that, but the sun, it will come. And then you have to have peace, and you have to accept it. And let it go. You know, it’s like, saying that, you get hurt, heal, but then, you’re going to get better. That’s what I tell them, always, no one will suffer for an entire year, or an entire life, it will always pass, you’re going to do better, and you’re going to accept it. You know, it’s hard because a lot of people, they’re hurt and they’re crying and I cried, but in my moment, but I don’t cry like I used to cry [at the start] when they told me I had breast cancer. That really hurt me. But then that passed, now I have to focus on my day to day and have peace, that’s what I tell people. You’re going to go through hard time, but it will pass.”
**P8**: “Probably because I was in shock with the news, they had given me. And I couldn’t understand the doctor, when she told me the type of cancer it was. But I have an appointment with her, or with them, this very week and I was thinking about asking them…”

## Data Availability

The datasets generated and analyzed during the current study are not publicly available in order to protect participant confidentiality but are available from the corresponding author on reasonable request.
